# A novel skin cancer detection model using modified finch deep CNN classifier model

**DOI:** 10.1038/s41598-024-60954-2

**Published:** 2024-05-16

**Authors:** Ashwani Kumar, Mohit Kumar, Ved Prakash Bhardwaj, Sunil Kumar, Shitharth Selvarajan

**Affiliations:** 1 Department of Computer Science and Engineering, School of Engineering and Technology, Sharda University, Greater Noida, India; 2 Department of Information Technology, School of Engineering, MIT-ADT University, Pune, 412201 India; 3 School of Computer Science, UPES, Dehradun, 248007 India; 4 Department of CSE, Galgotias College of Engineering & Technology, 1, Knowledge Park-II, Greater Noida, 201310 India; 5 Department of CSE, Kebri Dehar University, Kebri Dehar, Ethiopia

**Keywords:** Skin cancer disease, Deep CNN classifier, Feature extraction, Convergence, Modified Falcon finch, Ultraviolet radiation, Engineering, Electrical and electronic engineering

## Abstract

Skin cancer is one of the most life-threatening diseases caused by the abnormal growth of the skin cells, when exposed to ultraviolet radiation. Early detection seems to be more crucial for reducing aberrant cell proliferation because the mortality rate is rapidly rising. Although multiple researches are available based on the skin cancer detection, there still exists challenges in improving the accuracy, reducing the computational time and so on. In this research, a novel skin cancer detection is performed using a modified falcon finch deep Convolutional neural network classifier (Modified Falcon finch deep CNN) that efficiently detects the disease with higher efficiency. The usage of modified falcon finch deep CNN classifier effectively analyzed the information relevant to the skin cancer and the errors are also minimized. The inclusion of the falcon finch optimization in the deep CNN classifier is necessary for efficient parameter tuning. This tuning enhanced the robustness and boosted the convergence of the classifier that detects the skin cancer in less stipulated time. The modified falcon finch deep CNN classifier achieved accuracy, sensitivity, and specificity values of 93.59%, 92.14%, and 95.22% regarding k-fold and 96.52%, 96.69%, and 96.54% regarding training percentage, proving more effective than literary works.

## Introduction

Since according estimates on disease released by the American Cancer Society, the fatality rate for patients with skin cancer can reach 75%^1,2^ and the incidence of melanoma, which increases the fatality rate, is still rising at 14%^3^. Skin cancer is one of the cancer types with a high mortality rate and takes place throughout every phase of life^4^. Everywhere in the trillions of cells that make up the human anatomy, cancer can develop. In cancer, somebody cell divisions begin without being blocked, and these cells disseminate into the tissues around them. According to the needs of the body, human cells typically multiply and divide to create new ones^5,6^. The World Health Organization (WHO) estimates that such of all cancers diagnosed globally are skin cancers^7,8^. Dermatologists examine skin lesions for skin cancer, evaluate the patient's clinical data, and classify the lesions based on their experience^9,10^. Malignant and benign tumors are the two types of tumors that cancer can cause. Malignant tumors are those that primarily contain cancerous cells. Malignancy denotes a cell's ability to invade or spread to nearby tissues. Once a benign tumor is removed, it cannot return, whereas a malignant tumor can return after surgery. Although many benign tumours within many organs in the human body are normally not hazardous, the benign tumour in the brain can be dangerous to life^11^.

A confined, early-stage malignant skin tumor has a 99% 5-year survival rate, but if the tumor spreads to other body areas, the survival rate drops to 20%^12–14^. It can occasionally be found in a variety of dark hues. Moreover, it might appear in the skin as rose pink, azure, royal purple, or even be colorless^15,16^. Due to its rapid spread, it is more lethal and damaging. Melanoma can be found anywhere on the human body, despite the fact that all frequently manifests on the lower limb’s back^17^. Skin genetic risk factors can be reduced by detecting a person’s skin cancer at an early stage^18^. Several radar systems have been built for clinical application, including the diagnosis of breast cancer^19,20^, the accurate measurement of respiration^21,22^, the calculation of blood pressure^23–25^, and the detection of glucose levels^26,27^. The discrepancy between melanomas’ electrical characteristics and those of healthy skin tissues is how mm-wave radars identify skin cancer. Using theoretical modeling and experimental observations, the complicated permittivity of skin at micrometers frequencies is addressed. A pair of non-invasive in-package reflectometry working at 42 GHz and 70 GHz have in fact been used to diagnose tissue samples disorders such as slightly earlier skin malignancies^28^.

Deep learning methods significantly improve the performance of skin cancer detection and multiple imaging techniques are used for the detection more efficiently^29^. Few of the more well-liked technologies that have been suggested as alternatives to eye inspection of the disease and the automatic techniques are emerged^30–32^. As there are so many interference elements, such as keratin on the top layer of skin, substances utilized make skin lesions more visible, and unique disks used for additional identification, using dermoscopy images to actively monitor melanoma seems to be very difficult. Dermoscopy procedures are created to produce a clear image of the skin lesion, and then reflection is eliminated to improve the visual impact. Nevertheless, automatic skin lesion recognition might be challenging due to artifacts, skin color, low contrast, hairs^33^, veins, and comparable appearances of melanoma and non-melanoma^34,35^. The need for surveying of color features is growing in current days as it contains top-level visual features^36^. Dermoscopy employs polarized magnifying glass and incident light to identify skin surface characteristics. When compared to unaided observations, the rate of cancer detection using this method is greater. Nonetheless, the dermatologist's knowledge provides the sole factor that influences the detection's accuracy^37^. Due to the high prevalence and lack of specialists, the demand for computer-aided diagnostic (CAD) systems for malignancy has increased. Medical image analysis can now classify melanoma automatically due to recent developments in deep neural networks (DNNs)^10,38,39^. The need for surveying of color features is growing in current days as it contains top-level visual features.

### Key objectives

1. Multiple researches are available based on the skin cancer detection, however there still exists challenges in improving the accuracy, reducing the computational time. The major objective of this research is to accurately predict skin cancer using a modified falcon finch deep CNN classifier cancer in less stipulated time with reduced latency.
2. A novel skin cancer detection is performed using a modified falcon finch deep Convolutional neural network classifier (Modified Falcon finch deep CNN) that efficiently detects the disease with higher efficiency.
3. Tuning enhanced the robustness and boosted the convergence of the classifier that detects the skin cancer in less stipulated time. The modified falcon finch deep CNN classifier achieved accuracy, sensitivity, and specificity values of 93.59%, 92.14%, and 95.22% regarding k-fold and 96.52%, 96.69%, and 96.54% regarding training percentage, proving more effective than literary works.
4. This analysis is enabled for proving the superiority of the falcon finch deep CNN over the other existing methods and then comparative methods used in the research are K-Nearest neighbour, Decision Tree, Random Forest, Support Vector Classifier, Deep CNN, HHO deep CNN, SSA deep CNN.

The paper is organized in the manner as follows, the existing researches along with their novel methodologies, their advantages and disadvantages are described in Section “Literature review”. The novel framework developed in this research is explained detail in Section “Materials and methods” along with the mathematical modelling of the modified falcon finch optimization algorithm is also detailed. The output obtained by the model is described detail along with the parameter details in Section “Experimentation, results and analysis” and at last section “Conclusion and future scope” concludes the paper with future scope.

### Motivation

**Figure 1:**
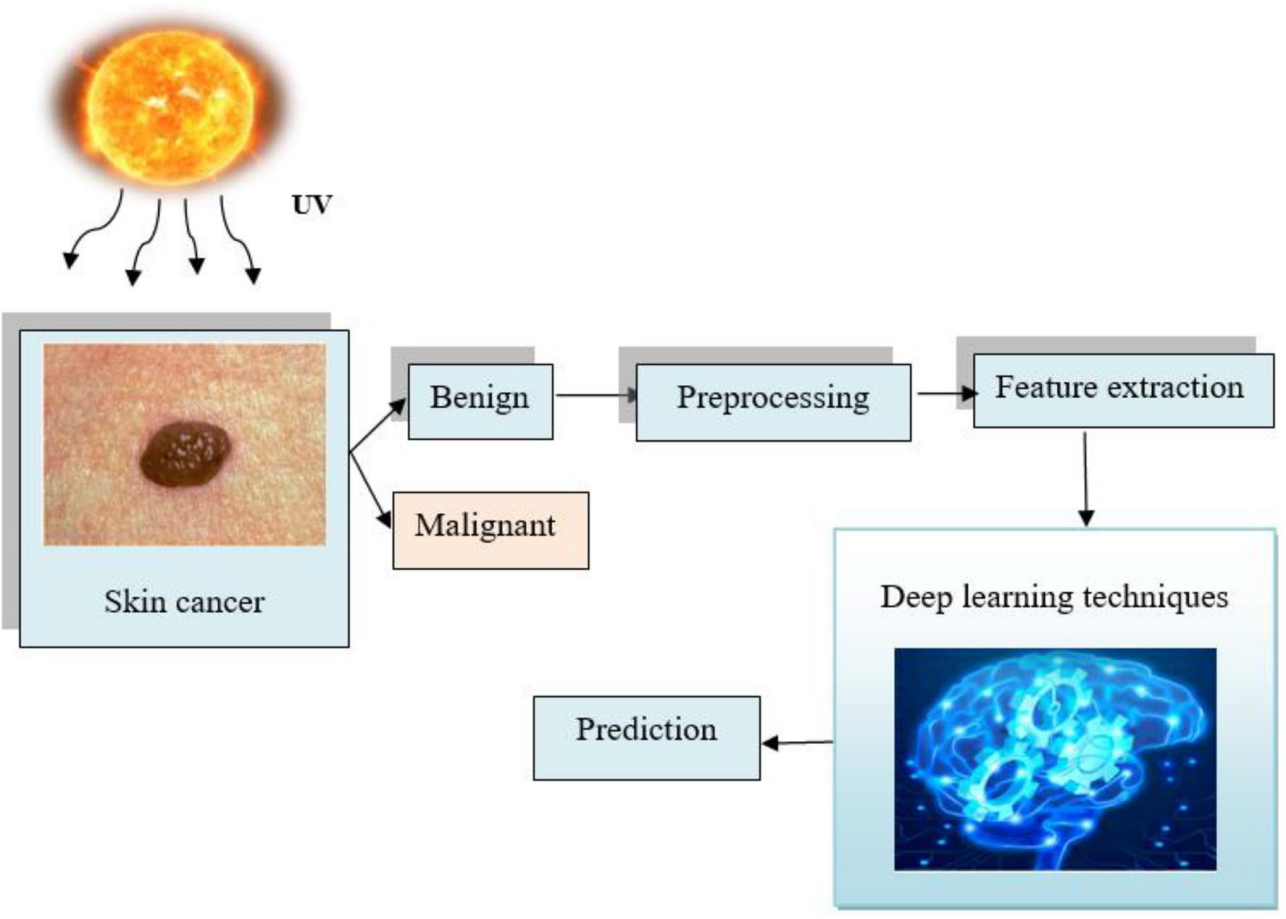
Framework for skin cancer detection.

Skin cancer is an emerging disease that are spreading in a higher rate and over 9500 people are diagnosed every day and is considered to be the 13^th^ most cancer occurs that occurs in men and 15th most cancer occurs in women. The impacts of this skin cancer include threatening of life as well as major financial losses. These impacts could be greatly reduced if the disease is identified at an earlier time. The existing methods possessed some difficulties due to high dimensional data, required large memory resources for the computation, asymmetry data, interclass variance, lack of information about training the classifier and so on. Hence there is a need for the development of novel framework for further improving the efficiency. Figure 1 schematically depicts the fundamental techniques developed in the diagnosis of skin cancer.

The major objective of this research is to accurately predict skin cancer using a modified falcon finch deep CNN classifier. The collected data is retrieved from the standard repository, and the aggregated data is kept in the device, where the corresponding users can access it. The preprocessing is done, and the characteristics required for skin cancer diagnosis are extracted, and then the modified falcon finch deep CNN classifier is used to detect the existence of skin cancer disease. The supremacy of the research is measured using the parameter metrics and is compared with the existing works. The significant contributions of the investigation are presented below.

- ***Falcon finch optimization algorithm:*** The Harris Hawk Optimization (HHO)^40^ and Sparrow Search Optimization (SSO)^41^ are frequently combined to create the falcon finch optimization. where the foraging characteristics of the finches are enhanced using the perching behavior of the falcon. The communication capability and the energy consumption of the finches is not efficient, which tends to fall to local optimum and is avoided by enhancing the communicative behavior from the falcons that helps in finding robust optimal solutions.
- ***Modified falcon finch deep CNN classifier****:* Utilizing the instructive characteristics either from images, the improved falcon finch deep CNN classifier efficiently diagnoses the existence of melanoma. By adjusting the hyper parameters of the deep CNN classifier using the falcon finch optimization algorithm, the efficiency is further increased.
- ***Feature extraction:*** The Resnet 101 and the statistical features helps in reducing the issues of over fitting.

## Literature review

The various literature works enabled based on skin cancer are reviewed and the observations are interpreted as follows, Pacheco and Krohling resolved the problems of composing images and extraction of the metadata features using a meta block that helps in the effect classification of skin cancer. The performance was improved due to the metadata enabled and provided better results in statistical test as well as balanced accuracy. The method provided more stability but there is a further need to improve the model to obtain more accurate results.

Wei et al. detected the lesions in the dermoscopy images using a lightweight network, where the features are effectively extracted using the CNN. A fusion strategy was applied to the extraction of the discriminative lesions that assist in enhanced performance. The method achieved high precision and the segmentation accuracy was also improved. Due to the limitation in the data, there is an excavation in extracting the discriminative features.

Ashraf et al. detected skin cancer by a novel framework developed relying on transfer learning. The method mainly focused on melanoma due to the fact that the mortality is high due to the cancer caused by melanoma. The method effectively identified the normal region and the melanoma-affected region, and the data imbalance issues were also avoided by enabling of the augmentation techniques. This method provided optimum results when the RoI was given, but when the full image was applied there is considerably low metrics values.

Khan et al.^42^ classified the skin lesion in the images and then the localization and segmentation was carried out. The binary images are fused with the layers in the 16-layered CNN model and then the segmentation was carried out using a contrast transform model. The redundancy in the model was eliminated by the downsampling. The method consumed more time for the recognition of the lesions, which could be reduced for further efficiency.

Hemsi et al.^43^ differentiated different kinds of moles and identified cancer using a deep learning framework. The method worked well even when there is heterogeneity and could able to detect from the retrained images. This method enhanced the detection performance and achieved lower false negatives. The method required fewer computational resources but initiated difficulties to resolve the issues of interclass variance.

Garg et al.^44^ detected and classified the skin cancer using the deep CNN, and the enabling of multiple augmentation techniques. The method eliminated the noise and improved the resolution of the image to a greater extent. The optimal training time and the augmentation of images effectively classified the disease. The research doesn’t provide a comprehensive interpretation of the training provided to the classifier.

Zhang et al.^45^ diagnosed the skin cancer using an optimized CNN and enabled the whale optimization for boosting the performance of the classifier. The difference between the network's actual response and the target value was lessened through multilayer perception modeling and gradient descent. The method provided better results but the comprehensibility should be further more needs enhancement.

Mansutti et al. A probe was designed that detected skin cancer in early stage. The dielectric substrate was used on the probe that provided accurate matching on the skin this can be used in the tool that can extract the correct affected images. This method is easily accessible due to cost, and mass production possibility. This method has more computational complexity.

Giulia et al. The skin lesions were detected using the global methods and the second system used a classifier names bag-of-features the color and texture features were used for lesion classification. This method achieved better results when color feature was used alone.

For skin cancer identification the identification of the epidermis area was important that is performed using watershed segmentation by Barata, C et al. from the segmented image GLCM and ABCD rules were used for feature extraction. Then the extracted features were classified using an SVM classifier. The SVM is non-linear and non-parametric hence the operation is low.

The boundaries of skin lesions from digital images were detected by Murugan et al.^46^ using partial-difference equations (PDE). Both anisotropic diffusion and contrast enhancement were used in preprocessing and the hair present in the images was removed using an preprocessing step called PDE-filter. This method is capable of covering a small area.

The skin lesion from different places was detected by Chung et al. using the colour histogram technique from dermoscopy images. This method included the capability of detecting the interior lesion regions.

Skin lesion detection involved two steps in Jaisakthi, et al. the illumination, hair, and rulers are removed using the segmentation technique called GrabCut algorithm. The color features are integrated with K-means clustering that improved the boundary segmentation and the Dice coefficient value is low.

A method was developed by Kumar et al. that classify the skin into cancerous and non-cancerous skin. The similar skin regions are separated using fuzzy C-means clustering. The image attribution was improved using the LBP and GLCM preprocessing techniques. The differential evolution algorithm was used in the ANN classifier and the parameter tuning takes more time.

The opposition-based learning and chaotic local search were combined with Harris Hawks optimization (HHO) by Pacheco and Krohling et al. that improved the performance of HHO that effectively solved the complication in feature selection. This does not cover a large area.

Lembhe et al.^47^ integrate machine learning and in-depth-based reading segmentation algorithms for detecting skin cancer at an early stage. However, need to add more techniques to improve the performance.

Viknesh et al.^48^ designed hybrid models such as SVM-based and CNN-based methods for detecting skin cancer, the classification accuracy achieved SVM classifier is 86.6%, while the CNN is 91% with 100 epochs. Table 1 shows the literature review of existing methods.

**Table 1:** Literature review of existing methods.

Sl. no	References	Approach	Advantages	Disadvantages	Achievements
1	Pacheco and Krohling ^10^	Metadata features using a meta block	Provided more stability	Need to improve the model to obtain more accurate results	Provided better results in statistical tests as well as balanced accuracy
2	Wei et al. ^3^	CNN	Extraction of the discriminative lesions that assist in enhanced performance	Due to the limitation in the data, there is an excavation in extracting the discriminative features	Achieved high precision and segmentation accuracy
3	Ashraf et al*.* ^18^	Novel framework	Effectively identified the normal region and the melanoma-affected region	When the full image was applied there are considerably low metric values	Provided optimum results when the RoI was given
4	Khan et al*.* ^42^	16- layered CNN model	Redundancy in the model was eliminated by the downsampling	Need to improve the segmentation accuracy. Time Complexity	–
5	Hemsi et al. ^43^	Deep learning framework	Required fewer computational resources	Resolve the issues of interclass variance	Achieved lower false negatives
6	Garg et al. ^44^	Deep CNN	Eliminated the noise and improved the resolution of the image to a greater extent	Doesn’t provide a comprehensive interpretation of the training	Achieved an accuracy of 91.6%
7	Zhang et al. ^45^	Optimized CNN	The target value was lessened through multilayer perception modeling and gradient descent	Comprehensibility should be furthermore needs enhancement	Achieved the best results and accuracy
8	Viknesh et al. ^48^	SVM-based and CNN-based methods	More accurate, and easier for non-dermatologists	Not suitable for larger datasets	The accuracy achieved SVM classifier is 86.6%, while the CNN is 91% with 100 epochs

## Research gaps

Effectively optimizing the classifier and choosing the desired solution within less stipulated time possess challenges. The research should identify the skin cancer in less stipulated time with reduced latency and achieving this is a strenuous task. Although dermoscopy is a non-invasive diagnostic method that uses optic magnification to enable the visualization of morphologic traits that are not visible to the naked eye, correct diagnosis is difficult and requires on sufficient training and expertise^2^. Data imbalance is a significant problem that can result in biased learning and have a detrimental effect on performance. The automatic detection of dermoscopy image lesions is difficult due to the complicated background and lesion features^3^. However, the higher model size also poses difficulties for future algorithmic applications; Eliminating over fitting problems is crucial since they have a direct impact on how accurately the malignant melanoma condition can be detected. While training lot of data there is a possibility of increase in the computational complexity of the classifier, which should be minimized to make it suitable for real time scenarios.

This research proposes a modified Falcon finch deep CNN classifier, which improves the classification accuracy and reduces the computational time compared to the existing methods. However, the quality of the input gets improved by the usage of preprocessing, and the Resnet- 101 and the statistical feature extractors are used to reduce the size of the feature vectors and the number of features in the feature vectors. Due to this, the time needed to train the model is reduced. Fine-tuning of hyperparameters in the classifier is provided by the proposed Falcon finch optimizer, and this improves the classification accuracy.

## Materials and methods

### Materials

#### Dataset

The input images are collected from the skin cancer detection dataset, which consists of 1800 images of benign moles and 1497 images of moles that have been classified as malignant. The goal of this kernel is to develop a model that can visually categorize a mole into benign and malignant. The dataset is drawn from the ISIC (International Skin Image Collaboration) Archive.

#### Data pre-processing

The dataset is developed based on the two classes such as benign and malignant. Initially the essential libraries are imported and then the pictures are loaded and the dictionary of images are labelled and then the categorization of these labels are performed. The normalization function is enabled for normalizing the data and then the training and testing data is split and then the cross validation will be performed.

#### Data augmentation

The new data is extracted from the already present training data and this process helps the data perfectly ready for the experiment by eliminating the overfitting problems, resizing, cropping, and reshaping. In this paper, these steps are elaborated in Sections “Data pre-processing”, and “Feature extraction”.

### Methods

The recent development in computer-aided diagnosis by analyzing medical images has become an advanced technique in both the technical field and medical fields. The proposed deep CNN is widely used because it reduces the image dimension without missing any of the details. Skin cancer is one of the diseases that cause high mortality, and the fine-grained variety in how skin lesions form makes it difficult to classify skin lesions automatically using images. The data collected from the standard repository consists of information of different patients, which is collected by connecting the IoT nodes to the patients. The collected information will be stored in the cloud for further preceding.

## Proposed methodology

Skin cancer is one of the diseases that cause high mortality, and the fine-grained variety in how skin lesions form makes it difficult to classify skin lesions automatically using images. The main aim of this research is to detect the skin cancer using modified falcon finch deep boosted deep CNN classifier availing the skin cancer detection dataset^49^.

### System model

**Figure 2:**
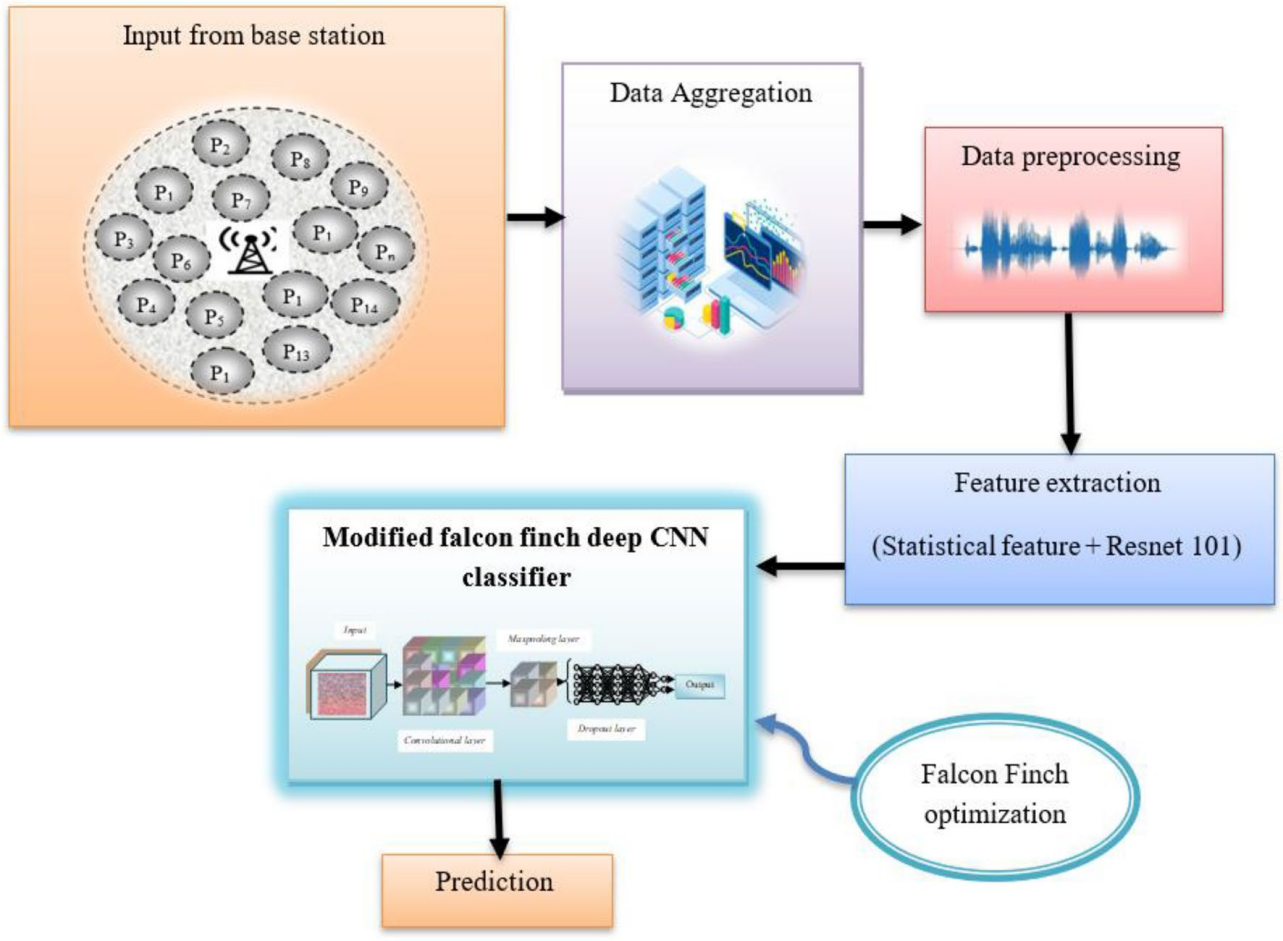
Skin cancer prediction model using modified falcon finch deep CNN classifier.

The base station serves as the network's hub, distributing data from many sources to the destination. Through a data aggregation method, the base station's huge pool of acquired data is condensed for high-level analysis. Preprocessing is done on the aggregated data after it has been obtained. Feature extraction is utilized to extract the features needed for the detection of skin cancer disease, while preprocessing improves image quality by removing unneeded noise. Figure 2 provides a systematic representation of the proposed skin cancer diagnosis model. The feature extraction is carried out by extracting the Resnet 101 feature, statistical features, and features, which are then fed forward to the modified falcon finch deep CNN classifier that successfully predicts the presence of the skin disease.

#### Input

The standard skin cancer detection dataset^49^ repository is used to gather the input image required for the identification of skin cancer. From the International Skin Image Collaboration Archive, the dataset is taken and the data is gathered from a variety of patients and stored in the base station, where communication is facilitated. The dataset contains a total of 3297 pictures, benign moles contain 1800 pictures and malignant classified moles contain 1497 pictures. All the pictures are resized to low resolution (224 × 224 × 3) RGB. The users can access the data from the base station whenever needed with proper access. The input data used in this research is mathematically formulated as follows,

1 $$ S = \left\{ {S_{1} ,\,S_{2} ,\,S_{3} ........S_{m} } \right\} $$

here, $S$ designates the skin cancer detection dataset and the number of images in the dataset is depicted by $m$.

#### Data aggregation

A big pool of data is summarized through the process of data aggregation in order to conduct in-depth analysis. At its most basic level, it entails gathering data from a variety of specified patients and organizing it into a more straightforward, and ease of use model and the aggregated data is represented using,

2 $$ S = \sum {S_{m}^{*} } $$

#### Data preprocessing

Fast NL Means Denoising preprocessing is utilized in this research which is used for suppressing noise, resizing and reshaping the image is performed in the preprocessing stage, which helps in improving the true quality of the image and is denoted by,

3 $$ S = \sum { Pre\left( {S_{m}^{*} } \right)} $$

#### Feature extraction

The raw image is transformed into the useful numerical features through extracting the features, which is necessary for the illness identification. Here the Resnet 101 feature and the statistical features are used for the detection and the dimension of the used features is $\left( {1 \times 109} \right)$.

## a) Resnet 101

Through an effort to alleviate the problems with gradient descent and to minimize the error, Resnet 101 features are extracted. Batch normalization is performed in the Resnet 101 that helps in normalizing the pixels and enhances the performance of the modified falcon finch deep CNN classifier.

## b) Statistical feature

The statistical features help in reducing the dimension of the features and the learning capability of the modified falcon finch deep CNN classifier is also boosted by the statistical features and is given by,

4 $$ F_{statistical} = \left\{ {\mu ,\,\sigma ,\,Var,\,Median,\,Skew,\,Kurt,\,H_{mean} ,\,G_{mean} } \right\} $$

***i) Mean:*** The mean value is evaluated by performing the sum of pixels to the total number of pixels present in the image.

5 $$ \mu = \frac{1}{q}\sum\limits_{x = 1}^{q} {Z_{x} } $$

***ii) Standard deviation:*** The measurement of the data's dispersion from the mean is measured and is given by,

6 $$ \sigma = \frac{1}{q}\sum\limits_{x = 1}^{q} {Z_{x}^{2} - \mu^{2} } $$

***iii) Variance:*** The measure of variability of the pixels present in the image is evaluated using the variance and is given by,

7 $$ Var = \frac{{\sum {\left( {Z_{x} - \mu } \right)^{2} } }}{q - 1} $$

***iv) Median:*** The determination of the center pixel present in the image is performed by median and is given by,

8 $$ Median = \left( {\frac{q + 1}{2}} \right) $$

***v) Skewness:*** The measurement of the assymetric distribution of the image is given by skewness, which is given by,

9 $$ Skew = \frac{x - \mu }{\sigma } $$

***vi) Kurtosis:*** Kurtosis measures how peaked or flat the data are in relation to a normal distribution and is formulated using,

10 $$ Kurt = \frac{{\mu^{4} }}{{\sigma^{4} }} $$

***vii) Harmonic mean:*** The color values of the pixels in the neighborhood replaces the color value of each pixel, which provides the relationship between the pixels and is given by,

11 $$ H_{mean} = q\left( {\sum\limits_{x = 1}^{q} {Z_{x} } } \right) $$

***viii) Geometric mean:*** The geometric mean enhances the smoothness of an image and helps in reducing the distortion caused by noise and is formulated by,

12 $$ G_{mean} = \left( {\mathop \Pi \limits_{x = 1}^{q} Z_{x} } \right) $$

here, $q$ denotes the number of images, and $Z$ denotes the individual image.

### Architecture and working of novel falcon finch deep CNN classifier

**Figure 3:**
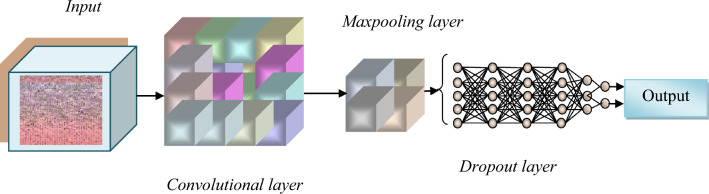
Architecture of falcon finch deep CNN classifier.

The modified falcon finch deep CNN classifier effectively analyzes the extracted features and helps in the prediction of the skin cancer. The extraction of high-level features helps in the robust determination and provides semantic information about the skin cancer. The CNN effectively extracts the features and converts the features to lower dimension without losing the originality. Figure 3 presents a schematic illustration of the modified falcon finch optimization.

#### Input layer

The falcon finch deep CNN classifier receives the characteristics obtained during the process of feature extraction as input.

#### Convolutional layer

The layer is made up of several filters, and one of those filters gathers all the data, convolves it, and then produces the feature map. The convolutional layer, which includes the classifier's weights and bias, lessens the issue of over fitting.

#### Maxpooling layer

The maximum values of the patches in the feature maps is determined using the maxpooling layer and performs down sampling operation. Training on a disproportionately of the small sample of the majority class samples is referred to as down sampling.

#### Dropout layer

Dropout layer is a mask that leaves all other neurons unaltered while eliminating particular neurons' contributions to the subsequent layer.

#### Fully connected layer

Each neuron in the simple layer of neurons known as the dense layer receives information from every cell in the layer below it. A dense layer is used to determine the presence of skin cancer based on the findings of the convolutional layers. The falcon finch optimizer is used to optimize the weights and bias in this layer, which speeds up convergence and uses less time. The parameter details of the deep CNN architecture are interpreted in Table 2.

**Table 2:** Parameters of modified falcon finch deep CNN classifier.

Layers	Output	Parameters
Conv 2D	(None, 22,11, 16)	160
Maxpooling 2D	(None, 22,11, 16)	0
Conv 2D_1	(None, 22,11, 32)	4640
Maxpooling 2D_1	(None, 22,11, 32)	0
Conv 2D_2	(None, 22,11, 64)	18,496
Maxpooling 2D_2	(None, 22,11, 64)	0
Dropout	(None, 22,11, 64)	0
Flatten	(None, 15,488)	0
Dense	(None, 64)	991,296
Conv 2D	(None, 22,11, 64)	18,496
Maxpooling 2D	(None, 22,11, 64)	0
Dropout	(None, 22,11, 64)	0
Flatten	(None, 15,488)	0
DenseS	(None, 64)	991,296
Dense_1	(None, 2)	130

### Falcon finch optimization

Generally, CNN has some limitations, the major limitation of CNN is accuracy increases the number of layers in the CNN also increases. Hence vanishing gradient problem occurs due to this the complexity increases. To avoid this problem introduced the optimization which tunes the hyperlayers of CNN. The falcon finch optimization is developed using the characteristics of falcon and finches, which is used for determining the optimal solution. The attributes in the deep BiLSTM classifier should be tuned efficiently for the reduction of error and improving accuracy and here the optimization is performed by enhancing the foraging characteristics of the finches. The foraging of finches takes place randomly and there is a possibility of falling into local optimum along with that the energy consumed for the foraging is also considerably high. When the perching behavior of the falcon is hybridized with the finches the energy consumption gets reduced, communication between the individuals is enhanced, which results in optimized output.

#### Inspiration of falcon finch optimization

The Falcon Finch Optimization (FFO) algorithm is initiated by mimicking the characteristic of finch (Sparrows) by availing their searching behavior that aids in determining the perfect solution. Normally, the foraging behavior of finch birds involves two categories of features that are producer and scrounger. The producer has a high level of energy for detecting food and providing information to all the scroungers for the foraging direction of food. When the finch bird detects the predator, it will make a chirp sound to alert everyone for moves to the safest place. The safe position is better than the previous position, the current position will be update it and gain more energy. The area inside with more energy they will acts as a producer, to achieve the best optimal value and improved the local optimum position of the scroungers. Similarly, the hawk variety that named as falcon which has an intelligent power for attaining the best food and to enhance the accurate results for obtained finch outcomes of global best optimal solution. These two characters are introduced for attained the best outcomes of operation to determine the target solution. Similarly, the Falcon (Harris Hawk) posses’ cooperative behavior with strong communication skills and the effecrtive perching behaviour, which helps in boosting the characteristics of finches.

#### Mathematical model of falcon finch optimization

The behavior of the finches is used for the adjusting of parameters in the classifier and the mathematical formulation of the behavior of finches is explained details in the below section.

##### Initialization stage

Initially, the population of the finches are initiated, which is used to determine the available solutions for the optimization and is mathematically represented by,

13 $$ Y_{fin} = \,\,\left[ {\begin{array}{*{20}c} {y_{1,1} } & {y_{1,2} } & \cdot & {y_{1,s} } \\ {y_{2,1} } & {y_{2,2} } & \cdot & {y_{2,s} } \\ \cdot & \cdot & \cdot & \cdot \\ {y_{l,1} } & {y_{l,2} } & \cdot & {y_{l,s} } \\ \end{array} } \right] $$

here, $l$ represents the count of finches in each position and $s$ represents the dimension of the variables to be optimized.

##### Fitness evaluation

After initiating the position, the fitness function of each finchis evaluated based on the below equation,

14 $$ H_{Y\,} = \,\,\,\left[ \begin{gathered} \,h\,\,\left( {\,\,\left[ {\begin{array}{*{20}c} {y_{1,1} } & {y_{1,2} } & \cdot & {\,\,\,\,\,y_{1,s} } \\ \end{array} } \right]\,} \right) \hfill \\ \,\,h\,\,\left( {\,\left[ {\begin{array}{*{20}c} {y_{2,1} } & {y_{2,2} } & \cdot & {\,\,\,y_{2,s} } \\ \end{array} } \right]\,} \right)\,\,\,\,\, \hfill \\ \,\,\,\,\begin{array}{*{20}c} \cdot & {\,\,\,\,\,\,\,\,\, \cdot } & {\,\,\, \cdot } & {\,\,\, \cdot } \\ \end{array} \,\,\, \hfill \\ \,h\,\,\,\left( {\,\left[ {\begin{array}{*{20}c} {y_{l,1} } & {y_{l,2} } & \cdot & {\,\,\,\,\,y_{l,s} } \\ \end{array} } \right]\,} \right) \hfill \\ \end{gathered} \right] $$

where, $H_{Y}$ represent the fitness value of finches and the best finch is evaluated based on the foraging behavior.

##### Objective function

The best finch that forages for food based on the perching behaviour is considered to be the objective function in this algorithm.

##### Foraging stage

In FFO algorithm the best finches are considered as producers and they are responsible for determining the food for the scroungers. The producers determine the food and guides other finches for obtaining the food. Hence the producers search for the food in a large search space and the position update of the producer finch is mathematically represented by,

15 $$ Y_{fin,\,i}^{k + 1} \, = \,\,\,\,\left\{ {\begin{array}{*{20}l} {Y_{fin,i}^{k} \cdot \exp \left( {\frac{ - i}{{\beta \cdot iter_{\max } }}} \right)} \hfill & {if\,T < P_{alarm} } \hfill \\ {Y_{fin,i}^{k} \cdot N_{rand} \cdot M} \hfill & {if\,T \, \ge {\text{P}}_{alarm} } \hfill \\ \end{array} } \right. $$

In the above equation, the current iteration is designated by $k$ in the dimension $i = 1,2, \ldots s$. $Y_{fin,i}^{k}$ denotes the $fin^{th}$ sparrow in the dimension $s$ during the iteration $k$.$\beta$ depicts the random number and is in the range $\left[ {0,1} \right]$, $N_{rand}$ denotes the random number that follows the normal distribution, $T$ represents a random number and depending upon the $T$ and alarm value $P_{alarm}$ the position update takes place and the value of $P_{alarm}$ is assigned to be $0.5$. $M$ denotes a matrix that consists of elements with the value $1$ in the dimension $1 \times s$.

##### Protective stage

The safety of the finches is evaluated based on two conditions described below:

$T < 0.5$: This designates that there are no predators around the finches and they are in the safest position.

$T \ge 0.5$: When the finches recognized a predator then this condition will occur and all the finches coordinatively moves towards a safer place away from the predators.

##### Accumulation stage

The scroungers obtain the food from the producers and again depending on the fitness function the best scrounger will obtain the food and other scroungers will wait for the next iteration for obtaining the food. The best scrounger continuously monitors for the food from the producers and when the producers appear the best scrounger suddenly fight and obtain the food. The position update of the scroungers is mathematically represented as,

16 $$ Y_{fin,i}^{k + 1} \, = \,\,\,\,\left\{ {\begin{array}{*{20}l} {N_{rand} \cdot \exp \left( {\frac{{Y_{worst}^{k} - Y_{fin,i}^{k} }}{{f^{2} }}} \right)} \hfill & {if\,\,fin > s/2} \hfill \\ {Y_{opt}^{k + 1} + \left| {Y_{fin,i}^{k} - Y_{opt}^{k + 1} } \right| \cdot B^{ + } \cdot M} \hfill & {otherwise} \hfill \\ \end{array} } \right. $$

where,$$Y_{opt}^{k + 1}$$ represents the optimal solution obtained by the producers, the current worst solution is represented by the $$Y_{worst}^{k}$$ designates the worst solution obtained during the iteration $$k$$ and $$B^{ + } = B^{T} \left( {BB^{T} } \right)^{ - 1}$$ and when the condition $$f > {\raise0.7ex\hbox{$s$} \!\mathord{\left/ {\vphantom {s 2}}\right.\kern-0pt} \!\lower0.7ex\hbox{$2$}}$$ then the $$f^{th}$$ scrounger is considered to be starving for the food.

The finding of the food by the scroungers is improved by using the soft and hard besiege of falcons. When enabling this soft and hard besiege the weakest scrounger also has the capability to determine the best food, which shows that the optimal solution could be determined even in local optimum and the two different cases are described below. Here $z$ is the parameter used to describe whether the scrounger is obtaining the food or not and the besiege of the finches is described by the parameter $b$. The value of $T$ denotes type of besiege to be taken by the scroungers.

**Case 1: **$$\left( {T \ge 0.5} \right)\& \& \left( {z \ge 0.5} \right)$$.

When the above condition is satisfied the soft besiege takes place and the scroungers will consume food through softbesiege and is expressed by,

17 $$ Y_{fin,i}^{k + 1} \, = \,\,\,\,\left\{ {\begin{array}{*{20}l} {x_{1} \left\{ {N_{rand} \cdot \exp \left( {\frac{{Y_{worst}^{k} - Y_{fin,i}^{k} }}{{f^{2} }}} \right)\,} \right\} + \,\,x_{2} \left( {Y_{scr}^{k} - Y_{fin}^{k} } \right) + x_{3} \left( {Y_{fin}^{k} - Y_{opt}^{k + 1} } \right);} \hfill & {if\,\,fin > s/2} \hfill \\ {\begin{array}{*{20}c} {Y_{opt}^{k + 1} } & + & {\left| {Y_{fin,i}^{k} - Y_{opt}^{k + 1} } \right| \cdot B^{ + } } \\ \end{array} \, \cdot \,M;} \hfill & {otherwise} \hfill \\ \end{array} } \right. $$

When the soft besiege occurs with rapid progressive dives then the next moves are evaluated using,

18 $$ M = \frac{1}{2}\left[ {Y_{scr}^{k} + T|V \cdot Y_{scr}^{k} - Y_{fin,i}^{k} | + Y_{avg}^{k} + \lambda \cdot \left( {Y_{fin,i}^{k} - Y_{opt}^{k} } \right)} \right] $$

here,$Y_{scr}^{k}$ denotes the scrounger in the $k^{th}$ iteration, $\lambda$ acts as an default constant that possess the value $0.5$, $x$ is a parameter used as an constant, $Y_{avg}^{k}$ represent the average of the individuals.

**Case 2:** $$\left( {T \ge 0.5} \right)\& \& \left( {z \ge 0.5} \right)$$.

When the above condition is satisfied the hard besiege takes place and the scroungers will consume food through hard besiege and is expressed by,

19 $$ Y_{fin,i}^{k + 1} \, = \,\,\,\,\frac{1}{2}\left\{ {Y_{scr}^{k} - E|\Delta Y_{fin}^{k} | + Y_{fin}^{k} + Q \cdot \left( {\frac{{|Y_{fin,i}^{k} - Y_{worst}^{k} |}}{{\left( {S_{best}^{k} - S_{worst}^{k} } \right)}}} \right)} \right\} $$

When the hard besiege occurs with rapid progressive dives then the next moves are evaluated using,

20 $$ M = \frac{1}{2}\left[ {Y_{scr}^{k} + \left( {1 + EV} \right) + Y_{fin}^{k} \left( {\lambda - E} \right) + Y_{avg}^{m} - Y_{opt}^{k} } \right] $$

##### Novel perching stage

The producer finches need lots of energy and time for foraging and although they feed the scroungers, the communication between the individual’s during foraging is considerably low, which results in poor global search ability. This could be overcome by integrating the perching characteristics of the falcon, because the falcon possesses the characteristics of identifying the prey by perching. The perching of the falcon takes place in two ways relying upon the presence of the family members of the falcons and the position of the prey. This reduces the prey's search duration, whereas perching helps the individual finches retain energy. These actions can be quantitatively expressed as follows:

Case 1:$$\left( {T < P_{alarm} } \right)\& \& \left( {p \ge 0.5} \right)$$.

When the safety threshold is met and the finches are in safer position and the position of the falcon is near to the family members then the perching will takes place based on the below equation,

21 $$ Y_{perch} = \,0.5\,Y_{fin,i}^{k} \cdot \exp \left[ {\frac{ - i}{{k_{\max } }}} \right] + \,0.5\,Y_{fal} \left( k \right) - n_{1} |Y_{fal} \left( k \right) - 2\,n_{2} \,Y_{curr} \left( k \right) $$

Case 2: $$\left( {T < P_{alarm} } \right)\& \& \left( {p < 0.5} \right)$$.

When the safety threshold is not met and the finches are surrounded by predator along with that position of the falcon is near to the food then the perching will take place based on the below equation, where the safety will also be maintained and at the same time the food.

22 $$ Y_{perch} = 0.5\left( {Y_{fin,\,i}^{k} + N_{rand} \cdot M} \right) + 0.5\left[ {Y_{prey} \left( k \right) - Y_{avg} \left( k \right) - n_{3} \left( {Z_{lb} + n_{4} \left( {Z_{ub} - Z_{lb} } \right)} \right)} \right] $$

where, $Y_{perch} \,$ designates the perching behavior, $Y_{fal}$ denotes the position of the falcon during the iteration $k$, $n_{1} ,\,n_{2} ,\,n_{3} ,\,n_{4}$ designates the random number in the range $\left( {0,\,1} \right)$. The position of the falcon is denoted by $Y_{fal}$ and during the current iteration the position will be represented by $Y_{curr} \left( k \right)$. $p$ denotes the perching behavior of the individuals and it provides the information about whether the falcon is near to the prey or family members.

##### Terminating stage

**Figure 4:**
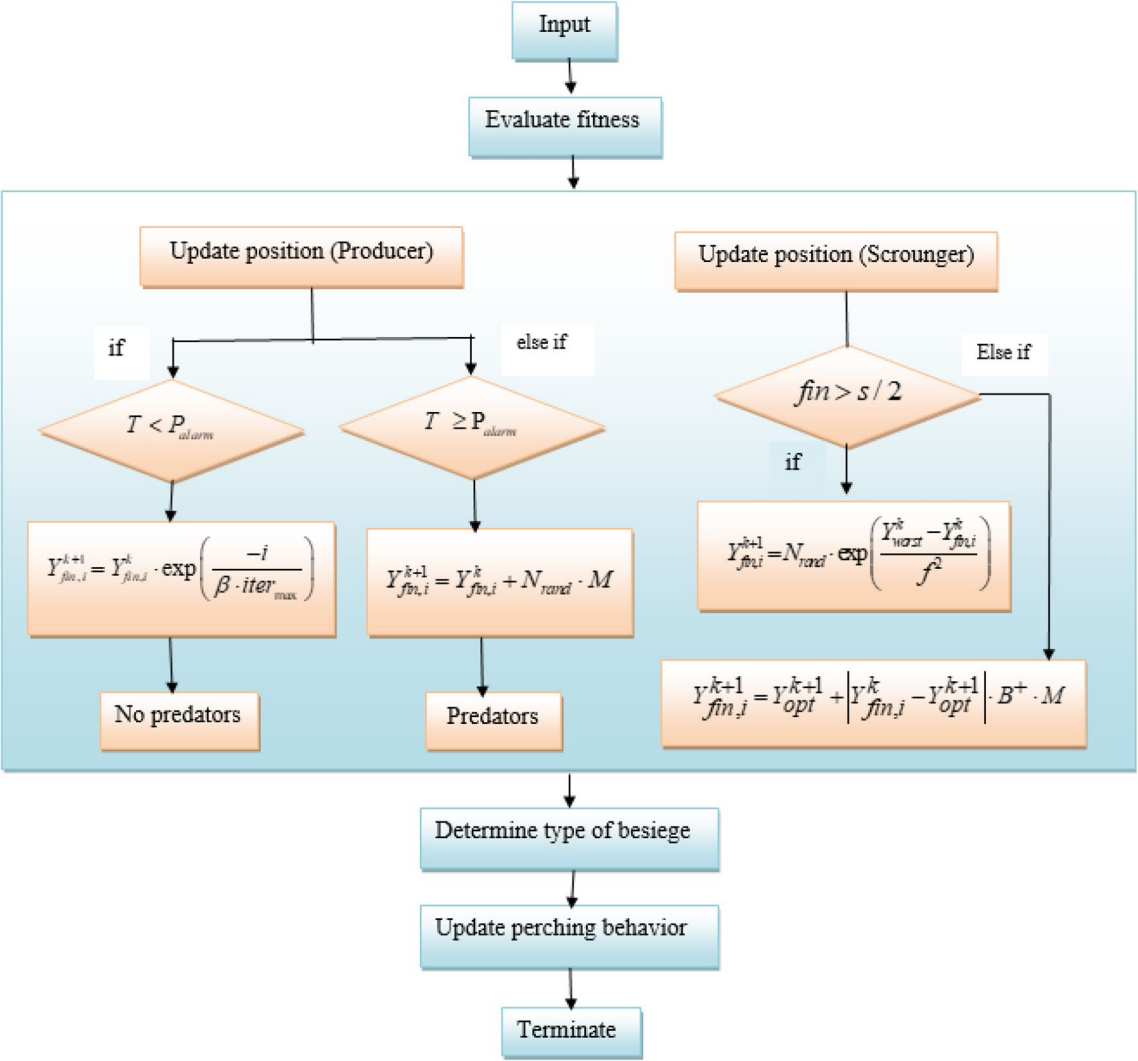
Flowchart for modified falcon finch optimization.

After determining the optimal solution, the process will gets terminated. The algorithmic steps in the falcon finch optimization is enumerated in algorithm 1 and schematically shown in Fig. 4.

**Algorithm 1:**
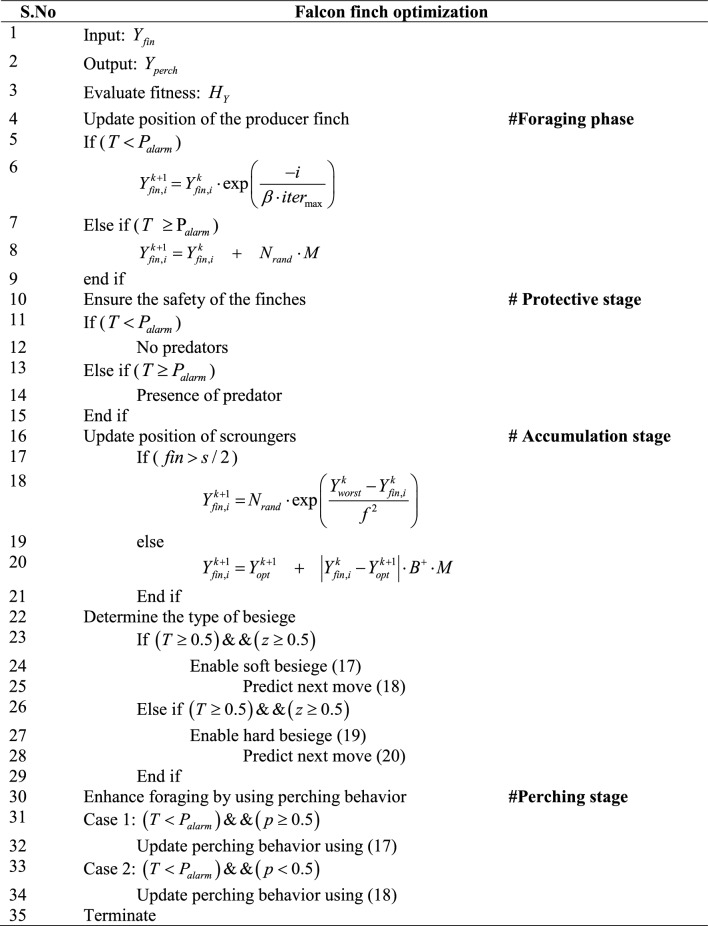
Pseudo code for falcon finch optimization.

## Experimentation, results and analysis

The sections that follow provide a thorough explanation of the findings made possible by the modified falcon finch deep CNN for skin cancer diagnosis.

### Experimental setup

The accuracy, sensitivity, and specificity of the study are measured to demonstrate the model's efficacy. The research is conducted using the PYTHON software on a Windows 10 computer with 8 GB of RAM. The hyperparameters of the network involve the batch size of 32, epochs of 100, default optimizer is adam, the loss is mse, and activation functions are relu and softmax.

### Performance metrices

The metrics used for measuring the improvement are measured concerning the k-fold and the training percentage and are described as follows,

**a) Accuracy:** The measure of how accurately the modified falcon finch deep CNN identified the skin cancer is measured using this metrics and is given by,

23 $$ Acc = \frac{{P_{t} + N_{t} }}{{P_{t} + N_{t} + P_{f} + N_{f} }} $$

**b) Sensitivity:** The ratio of the number of instances that are correctly identified by the modified falcon finch deep CNN to the average of the correctly identified and wrong predicted instances and is given by,

24 $$ Sen = \frac{{P_{t} }}{{P_{t} + N_{f} }} $$

**c) Specificity:** The ratio of the number of instances that are misidentified by the modified falcon finch deep CNN to the average of the misidentified and correctly predicted instances and is given by,

25 $$ Spec = \frac{{N_{t} }}{{N_{t} + P_{f} }} $$

### Performance analysis

The performance of the falcon finch deep CNN is quantitatively evaluated using the performance analysis and is performed concerning both k-fold and training percentage and the observations are shown below. The performance of the falcon finch deep CNN is improved for the epoch 100 to greater extent. Here the utility of one epoch shows that the entire data is processed for a single time and for obtaining an optimal solution several epochs are needed. During the 100^th^ epoch the optimal solution is obtained.

#### Performance evaluation concerning k-fold

**Figure 5:**
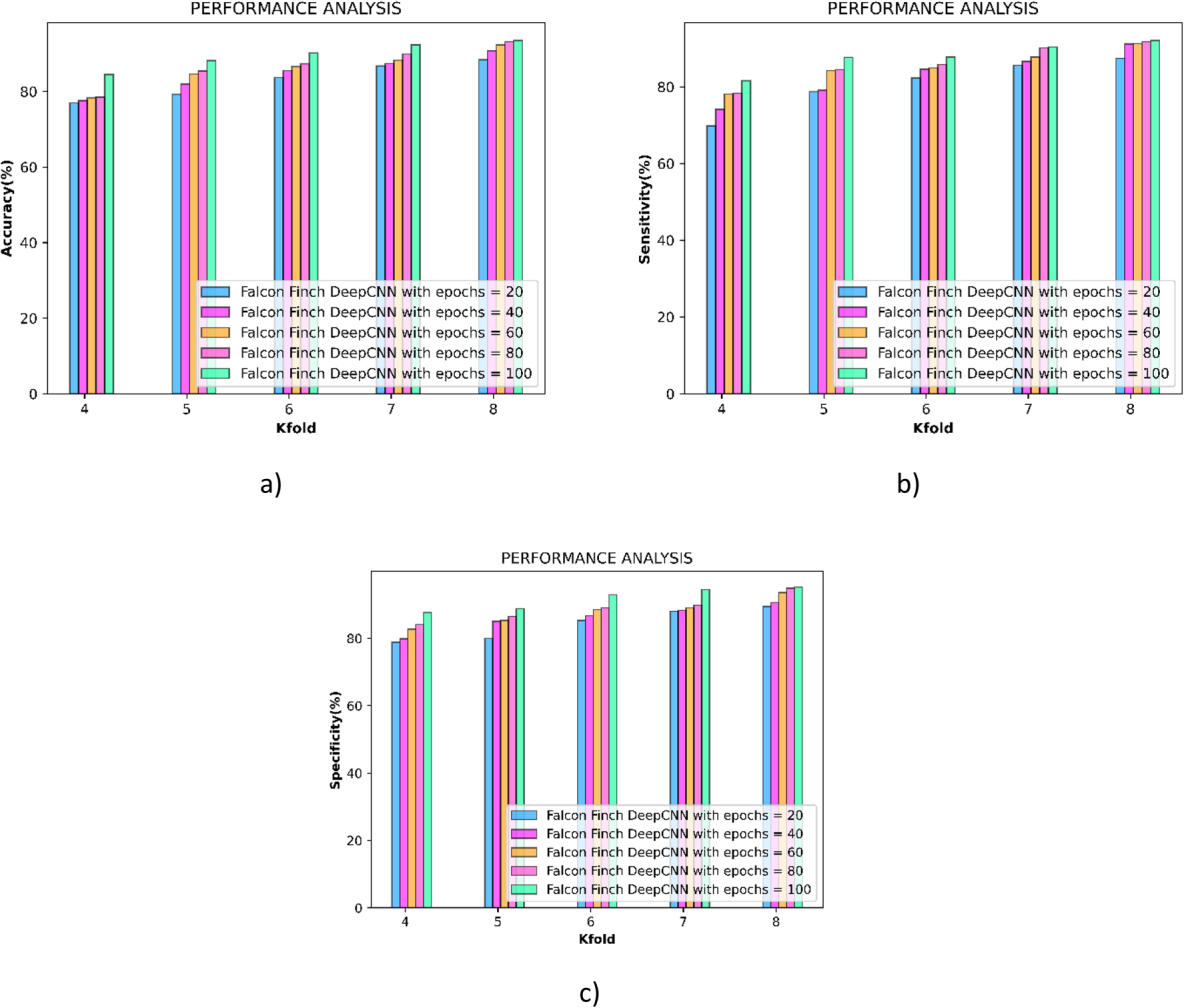
Performance analysis concerning k-fold (**a**) accuracy (**b**) sensitivity (**c**) specificity.

Figure 5 depicts the performance achieved by the falcon finch deep CNN during the epochs 20, 40, 60, 80 and 100. Initially, the metrics accuracy is measured and the outcomes are presented in the Fig. 5a, and the falcon finch deep CNN obtained the values of 88.39%, 90.82%, 92.32%, 93.22%, 93.59% during the k-fold 8. Correspondingly the sensitivity of the falcon finch deep CNN obtained the values of 88.39%, 87.45%, 91.20%, 91.26%, 91.77%, 92.14% during the k-fold 8 shown in Fig. 5b. At last, the sensitivity is measured and the falcon finch deep CNN achieved the values of 89.50%, 90.61%, 93.57%, 94.86%, 95.22% for k-fold 8 and is interpreted in Fig. 5c.

#### Performance evaluation concerning training percentage

**Figure 6:**
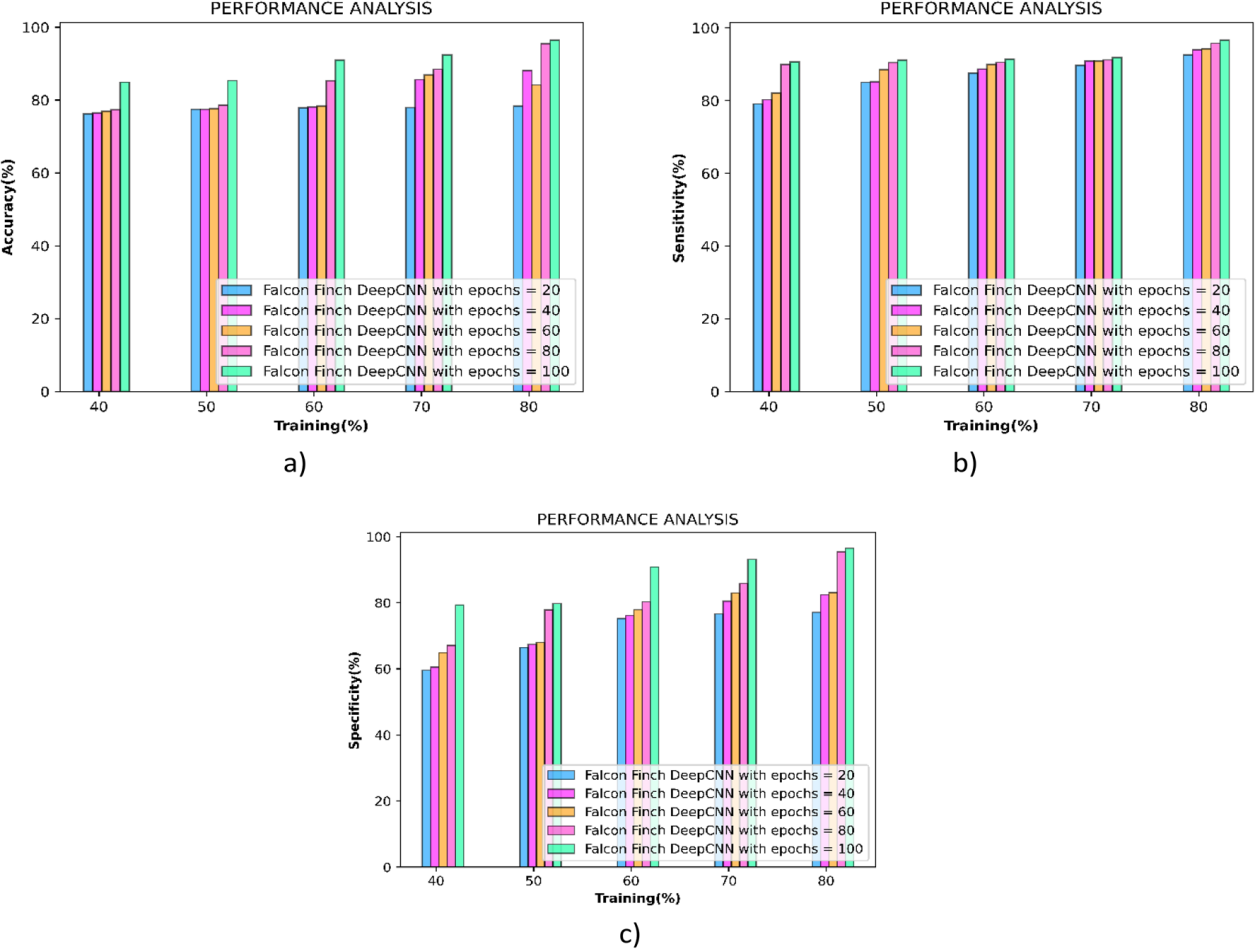
Performance analysis concerning training percentage (**a**) accuracy (**b**) sensitivity (**c**) specificity.

Figure 6 depicts the performance achieved by the falcon finch deep CNN during the epochs 20, 40, 60, 80 and 100. Initially, the metrics accuracy is measured and the outcomes are presented in the Fig. 6a, and the falcon finch deep CNN obtained the values of 78.40%, 88.15%, 84.16%, 95.52%, 96.52% during the k-fold 8. Correspondingly the sensitivity of the falcon finch deep CNN obtained the values of 92.58%, 94.03%, 94.27%, 95.77%, 96.69% during the k-fold 8 shown in Fig. 6b. At last, the sensitivity is measured and the falcon finch deep CNN achieved the values of 77.12%, 82.45%, 83.10%, 95.45%, 96.54% for k-fold 8 and is interpreted in Fig. 6c the performance analysis is tabulated in Table 3.

**Table 3:** Performance analysis of proposed method using k-fold and Training percentage.

Epoch	Accuracy	Sensitivity	Specificity
K-Fold-8			
20	88.39	87.45	89.50
40	90.82	91.20	90.61
60	92.32	91.26	93.57
80	93.22	91.77	94.86
100	93.59	92.14	95.22
Training percentage			
20	78.40	92.58	77.12
40	88.15	94.03	82.45
60	84.16	94.27	83.10
80	95.52	95.77	95.45
100	96.52	96.69	96.54

### Comparative analysis

This analysis is enabled for proving the superiority of the falcon finch deep CNN over the other existing methods and the comparative methods used in the research are K-Nearest neighbour^47^, Decision Tree^50^, Random Forest^47^, Support Vector Classifier^51^, Deep CNN^43^, HHO deep CNN^52^, SSA deep CNN^53^.

#### Comparative analysis concerning k-fold

**Figure 7:**
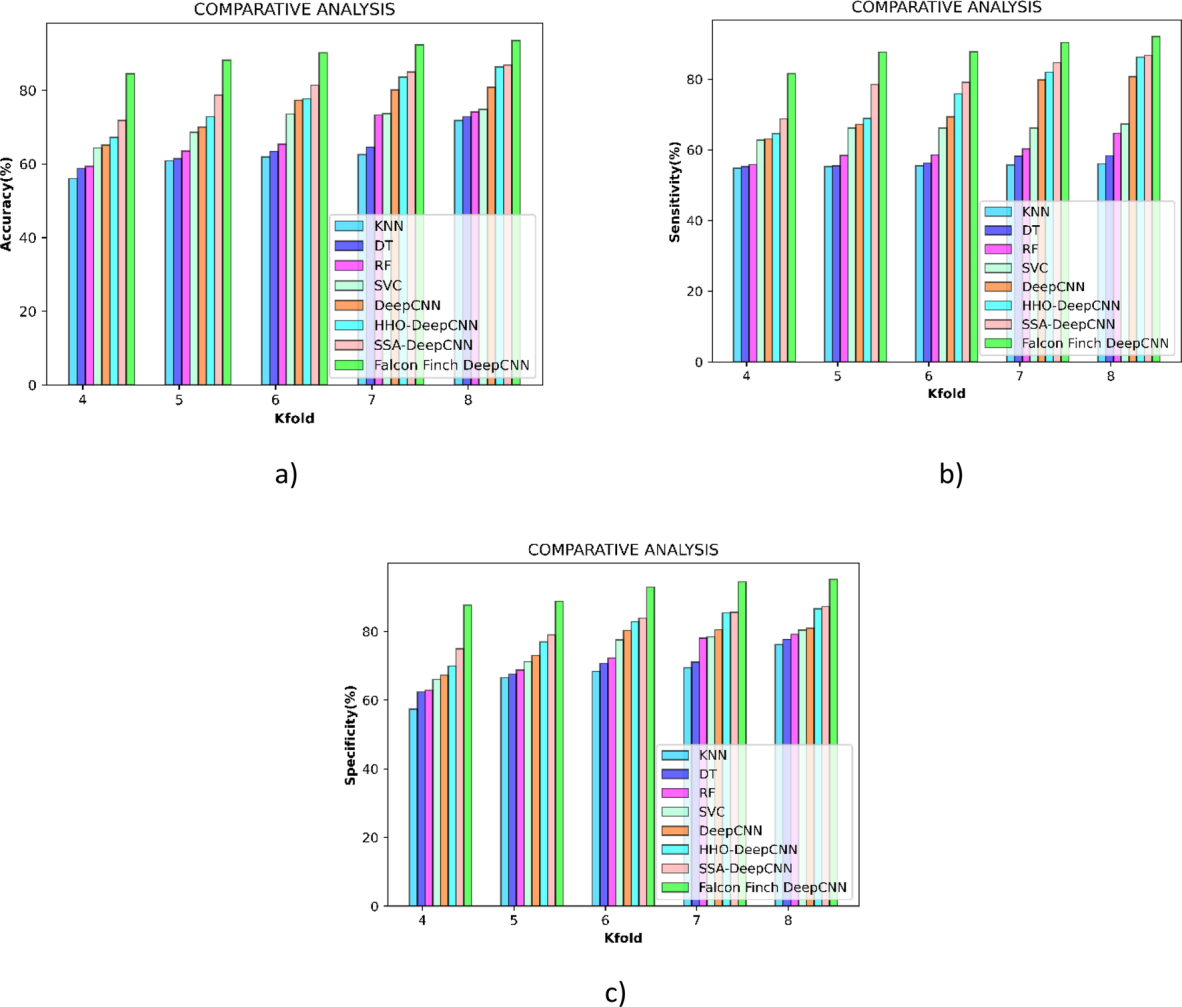
Comparative analysis concerning k-fold (**a**) accuracy (**b**) sensitivity (**c**) specificity.

Figure 7a shows the comparative performance in terms of metrics accuracy; during the k-fold 8 it outperformed SSA deep CNN by 7.16%.

Figure 7b interprets the relative performance in terms of metrics sensitivity, and throughout the k-fold 8 it improves by 6.42% in comparison to HHO deep CNN.

Figure 7c enumerates the relative performance in terms of metrics sensitivity. During the k-fold 8 comparison with deep CNN, an improvement of 14.954% is made by the modified falcon finch deep CNN classifier. The values obtained are interpreted in Table 4.

**Table 4:** Comparative analysis concerning k-fold.

Methods/K-fold	KNN	DT	RF	SVC	Deep CNN	HHO deep CNN	SSA deep CNN	Proposed
**4**	56.02	58.76	59.31	64.37	65.11	67.23	71.83	84.56
**5**	60.87	61.49	63.51	68.65	70.05	72.85	78.69	88.15
**6**	61.89	63.43	65.38	73.56	77.30	77.73	81.41	90.23
**7**	62.55	64.59	73.31	73.70	80.07	83.60	85.04	92.35
**8**	71.76	72.83	74.15	74.81	80.78	86.36	86.88	93.59
4	54.76	55.23	55.85	62.80	63.08	64.65	68.81	81.57
5	55.32	55.53	58.40	66.18	67.22	68.87	78.51	87.67
6	55.48	56.28	58.58	66.20	69.33	75.87	79.13	87.71
7	55.76	58.20	60.31	66.21	79.77	82.00	84.70	90.40
8	56.05	58.34	64.69	67.32	80.75	86.22	86.72	92.14
**4**	57.38	62.42	62.89	66.07	67.28	69.95	75.00	87.71
**5**	66.54	67.58	68.75	71.25	73.03	76.97	79.03	88.80
**6**	68.43	70.70	72.32	77.52	80.27	82.90	83.86	92.92
**7**	69.45	71.11	78.09	78.40	80.52	85.38	85.55	94.48
**8**	76.19	77.72	79.16	80.41	80.98	86.66	87.22	95.22

#### Comparative analysis concerning training percentage

**Figure 8:**
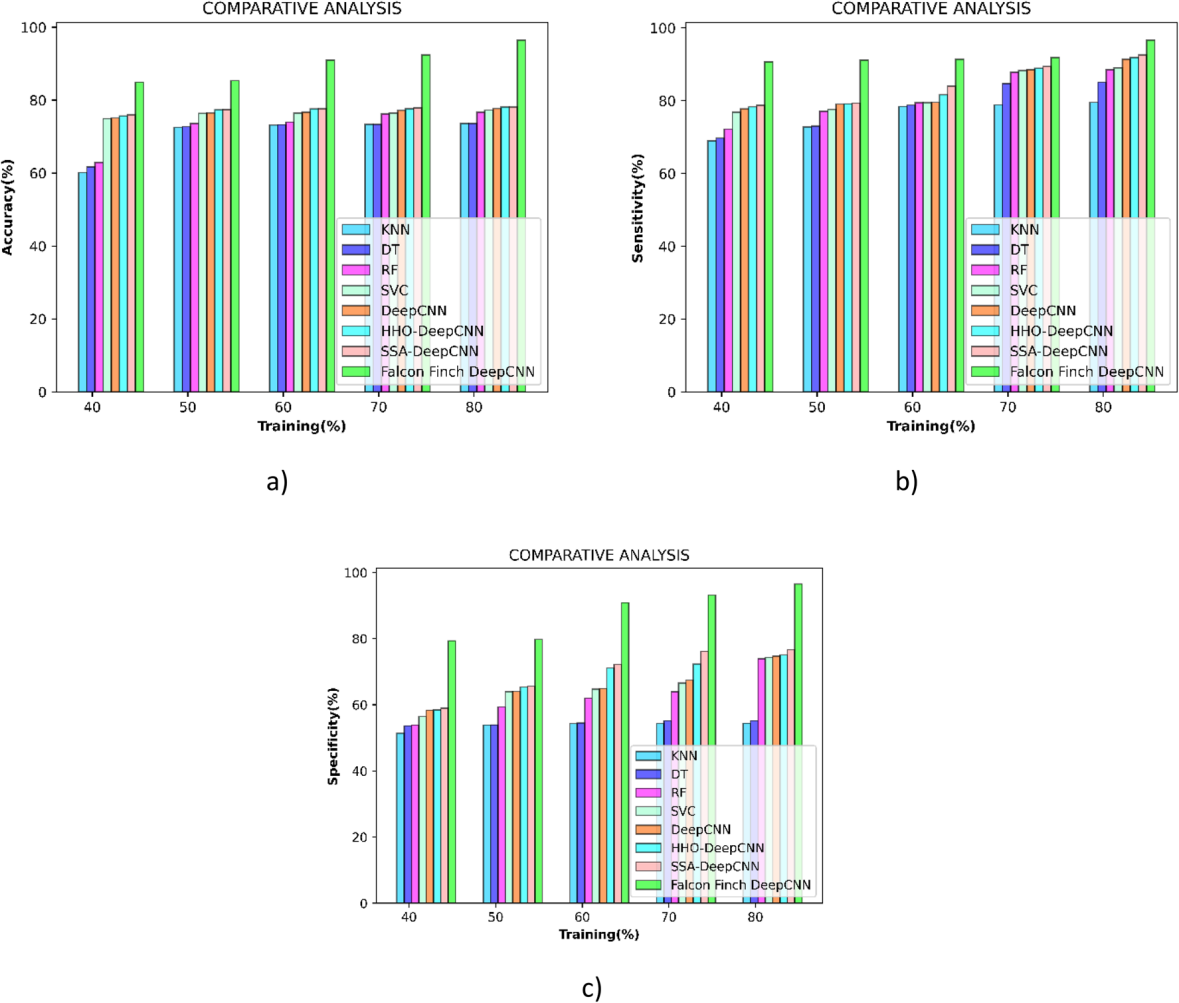
Comparative analysis concerning training percentage (**a**) accuracy (**b**) sensitivity (**c**) specificity.

Figure 8a shows the comparative performance in terms of metrics accuracy, and at training percentage 80, it improves by 19.05% when compared to SSA deep CNN.

As compared to HHO deep CNN during the k-fold 8, Fig. 8b shows the comparative performance in terms of metrics sensitivity, and an improvement of 4.91% is made by the modified falcon finch deep CNN classifier.

Figure 8c shows the performance comparison in terms of metrics sensitivity, and during the eighth k-fold, a 22.65% improvement over deep CNN is made modified falcon finch deep CNN classifier. The values obtained are interpreted in Table 5.

**Table 5:** Comparative analysis concerning training percentage.

Methods/Training percentage	KNN	DT	RF	SVC	Deep CNN	HHO deep CNN	SSA deep CNN	Proposed
40	60.12	61.64	62.96	74.96	75.10	75.67	75.96	84.93
50	72.56	72.75	73.59	76.38	76.52	77.35	77.42	85.38
60	73.21	73.27	73.99	76.41	76.70	77.57	77.68	91.02
70	73.35	73.39	76.24	76.44	77.23	77.66	77.94	92.42
80	73.56	73.59	76.65	77.26	77.72	78.10	78.13	96.52
40	68.94	69.76	72.19	76.83	77.70	78.34	78.71	90.68
50	72.77	73.01	77.07	77.60	79.06	79.13	79.35	91.17
60	78.39	78.80	79.48	79.50	79.55	81.67	84.05	91.35
70	78.85	84.72	87.83	88.31	88.48	88.92	89.40	91.86
80	79.55	85.05	88.45	89.06	91.36	91.85	92.52	96.69
40	51.42	53.64	53.86	56.44	58.32	58.45	58.97	79.35
50	53.88	53.92	59.35	64.01	64.04	65.35	65.59	79.75
60	54.34	54.47	62.03	64.75	64.87	71.13	72.22	90.88
70	54.34	55.19	63.94	66.59	67.47	72.28	76.20	93.16
80	54.41	55.19	73.95	74.35	74.67	75.06	76.69	96.54

### Analysis and discussion

A certain section compares and contrasts the deep CNN classifier for the modified falcon finch. The various state of art methods used for the skin cancer detection are interpreted with their metrics values and the values shows that the modified falcon finch deep CNN classifier is more efficient compared to the existing works. The improvement achieved is due to the enabled falcon finch optimization, where the falcon’s perching behavior is used to improve the foraging abilities of the finches. By improving the finches' inefficient communication and energy use, which has a tendency to fall to local optimum, the communicative behavior of the falcons, which aids in the discovery of reliable optimal solutions. The comparative discussion of the falcon finch deep CNN is interpreted in Table 6.

**Table 6:** Comparative discussion.

Methods	K-fold			Training percentage		
	Accuracy	Sensitivity	Specificity	Accuracy	Sensitivity	Specificity
KNN	71.76	56.05	76.19	73.56	79.55	54.41
DT	72.83	58.34	77.72	73.59	85.05	55.19
RF	74.15	64.69	79.16	76.65	88.45	73.95
SVC	74.81	67.32	80.41	77.26	89.06	74.35
Deep CNN	80.78	80.75	80.98	77.72	91.36	74.67
HHO deep CNN	86.36	86.22	86.66	78.10	91.85	75.06
SSA deep CNN	86.88	86.72	87.22	78.13	92.52	76.69
Proposed	93.59	92.14	95.22	96.52	96.69	96.54

## Conclusion and future scope

The skin cancer detection using the modified falcon finch deep CNN is performed in this research. These techniques are primarily appropriate for real-time medical applications, particularly in dermatology. A modified falcon finch deep CNN, which diagnoses the disease more effectively, is used in this study. With the modified falcon finch deep CNN classifier, it was possible to analyze the data pertinent to skin cancer while minimizing errors. The inclusion of the falcon finch optimization in the deep CNN classifier was significant because it provided the foraging and perching traits necessary for efficient parameter tuning. With this adjustment, the classifier can identify skin cancer in a shorter duration with more robust and improved convergence. By comparing the evaluation metrics, it can be seen that the research is more effective than previous studies in that the modified falcon finch deep CNN classifier achieved accuracy, sensitivity, and specificity values of 93.59%, 92.14%, and 95.22% for k-fold and 96.52%, 96.69%, and 96.54% for training percentage. However, the visual examination is complicated as the benign and malignant resemble the same in appearance for similar categories of lesions due to the complex structure that increases the complexity of the analysis. Scalability issues arise with larger datasets which consumes more hardware requirements for the implementation of the model that should be minimized in the future. In future the skin cancer detection and classification will be performed with hybrid classifiers. The non-melanoma skin cancer and melanoma cancer can be classified by further researchers in order to provide a comprehensive work relevant to skin cancer disease.

## Data Availability

The dataset used during the current study are available on reasonable request from the first author.

## Code availability

In order to enhance reproducibility, we have uploaded our implementation code on GitHub link: GitHub Repository. The repository includes scripts for data preprocessing and evaluation. We invite researchers to leverage and expand upon our codebase for experimental purposes and to advance our findings.
